# Practitioners, Friendly Societies, and the Bill

**Published:** 1911-07-08

**Authors:** J. P. Westrup

**Affiliations:** Fisherton House, Salisbury.


*To the Editor of* The Hospital.

### PRACTITIONERS, FRIENDLY SOCIETIES, AND THE BILL.

*To the Editor of* The Hospital.

Sir,—I was surprised to read the articles on the above Bill in your issues of June 10 and July 1.

I take great exception to them. Apparently you do not say a word against the "Friendly Societies." If you had to work as one of their medical officers, I venture to say you would write in a different tone.

I agree with Sir Henry Craik, namely, that one of the chief points is the behaviour of the Friendly Societies to the medical profession, and that we must fight them.

How would a solicitor like to be at the beck and call of a client for twenty-four hours of every day in the year for 6s.? And we have to do it for 4s.!

My remarks do not refer to a society like the National Deposit Friendly Society. They pay for work done—a very great point. Their members also have to pay their proportion according to the amount of the medical attendance—a healthy deterrent to unnecessary medical calls.

I will illustrate how some of the Friendly Societies treat their medical officers. A grocer, in order to facilitate the selling of 2 lb. of tea, throws in a pound of sugar. In the Friendly Societies the pound of sugar represents the doctor.

Many a club patient sends for a medical man because he can do so without paying for the attendance; if he had to pay only a very small fee for the visit he would not send for the doctor nearly so often.

Let me commend to your notice an excellent article on "Doctors and the Bill" in the "Spectator" for June 24, and to an equally good leading article on Malingering in the "Times" for June 29.

As regards the Bill, among the chief points are these: (1) A wage-limit of 30s.; (2) doctors to be paid for work done; (3) doctors to be adequately paid, having due regard for night visits, mileage, Sunday work, etc.; (4) doctors to be quite independent of Friendly Societies and their sweating ways.

I have attended the deserving poor for nothing and it has been a pleasure to me.—Yours faithfully,

Fisherton House, Salisbury. J. P. Westrup.

July 2.

